# Campanacci Grade III Giant Cell Tumor of the Distal Femur Treated With Wide En Bloc Resection and Cemented Modular Megaprosthetic Reconstruction: A Case Report

**DOI:** 10.7759/cureus.116361

**Published:** 2026-09-16

**Authors:** David Baudilio Leal Robles, Sebastian Alejandro Verduzco-Sierra, David De Jesus Garza-Villarreal, Othoniel Cruz-Jimenez

**Affiliations:** 1 Orthopaedics and Traumatology, Instituto de Seguridad y Servicios Sociales de los Trabajadores del Estado (ISSSTE) Hospital Regional Monterrey, Monterrey, MEX; 2 Pathology, Instituto de Seguridad y Servicios Sociales de los Trabajadores del Estado (ISSSTE) Hospital Regional Monterrey, Monterrey, MEX

**Keywords:** campanacci, distal femur, en bloc resection, gctb, limb salvage, megaprosthetic reconstruction

## Abstract

Giant cell tumor of bone (GCTB) is a benign but locally aggressive primary bone neoplasm that accounts for approximately 5% of all primary bone tumors and 20% of benign skeletal neoplasms. It most commonly presents between the second and fourth decades of life and typically involves the epimetaphyseal region of long bones in skeletally mature patients. Campanacci grade III tumors exhibit extensive cortical destruction and soft-tissue extension, which may preclude joint-preserving intralesional treatment.

We report the case of a 26-year-old woman with a two-year history of progressive left knee pain, distal thigh swelling, and functional limitation. Radiographs and computed tomography revealed an expansile osteolytic lesion of the distal femur with extensive cortical destruction. Histopathological examination following an open biopsy confirmed GCTB, radiographically classified as Campanacci grade III. Given the extent of cortical and articular involvement, limb-salvage surgery was performed through wide en bloc resection of the distal femur, followed by reconstruction with a cemented modular distal femoral megaprosthesis. Rehabilitation was initiated on the third postoperative day, and no immediate postoperative complications occurred. At 12 months, the patient had a Musculoskeletal Tumor Society (MSTS) score of 26/30 (87%), substantial pain relief, and return to daily activities. Serial radiographs demonstrated stable prosthetic alignment and fixation without mechanical failure or aseptic loosening. No local recurrence or distant metastasis was identified.

Wide resection followed by megaprosthetic reconstruction can provide satisfactory short-term oncologic and functional outcomes in selected patients with Campanacci grade III GCTB of the distal femur when extensive bone and joint destruction makes joint preservation unsuitable. Longer follow-up remains necessary to assess disease recurrence and implant survival.

## Introduction

Giant cell tumor of bone (GCTB) is a benign but locally aggressive primary bone neoplasm characterized by osteoclast-like multinucleated giant cells within a mononuclear stromal cell background. It accounts for approximately 5% of primary bone tumors and 20% of benign skeletal neoplasms, with peak incidence between the second and fourth decades of life. Despite its benign histology, GCTB can cause progressive bone destruction, cortical breakthrough, soft-tissue extension, pathological fracture, local recurrence, and, rarely, pulmonary metastasis [1,2].

The distal femur is among the most frequently affected sites, together with the proximal tibia and distal radius. Diagnosis integrates clinical evaluation, radiography, cross-sectional imaging, and histopathological confirmation. The Campanacci system grades radiographic aggressiveness and informs surgical planning [3]. Grade III lesions demonstrate cortical destruction and extraosseous extension and generally require more aggressive treatment than lower-grade tumors.

Treatment aims to achieve local tumor control while preserving limb function and minimizing recurrence. Extended intralesional curettage is preferred for most grade I and II lesions, whereas wide en bloc resection may be required for grade III tumors with extensive cortical destruction, articular involvement, or soft-tissue extension [4-6]. Endoprosthetic reconstruction can restore stability and facilitate early rehabilitation after the resection of large periarticular defects. We present a Campanacci grade III GCTB of the distal femur treated with wide resection and distal femoral megaprosthetic reconstruction, emphasizing the rationale for limb-salvage surgery and the early functional and oncologic outcomes.

## Case presentation

A 26-year-old woman with no significant medical history was referred with a two-year history of progressively worsening left knee pain, distal-thigh swelling, and functional limitation. She denied trauma, fever, constitutional symptoms, or recent weight loss. The pain increasingly limited ambulation and activities of daily living. Examination revealed a firm, well-circumscribed swelling over the anteromedial distal femur with localized tenderness. Knee motion was limited from 0 degrees of extension to 80 degrees of flexion because of pain. The overlying skin was intact, and distal neurovascular findings were normal.

Diagnostic assessment

Anteroposterior and lateral radiographs demonstrated an expansile osteolytic lesion of the distal femoral metaphysis with cortical thinning and disruption, raising concern for an aggressive primary bone tumor (Figure 1). Computed tomography showed a well-defined expansile lytic lesion extending from the distal metaphysis toward the epiphysis, with marked cortical thinning, focal posterior cortical destruction, and extension to the subchondral bone; no matrix mineralization or pathological fracture was identified (Figure 2). Chest radiography and thoracoabdominal computed tomography showed no pulmonary or visceral metastases.

**Figure 1:**
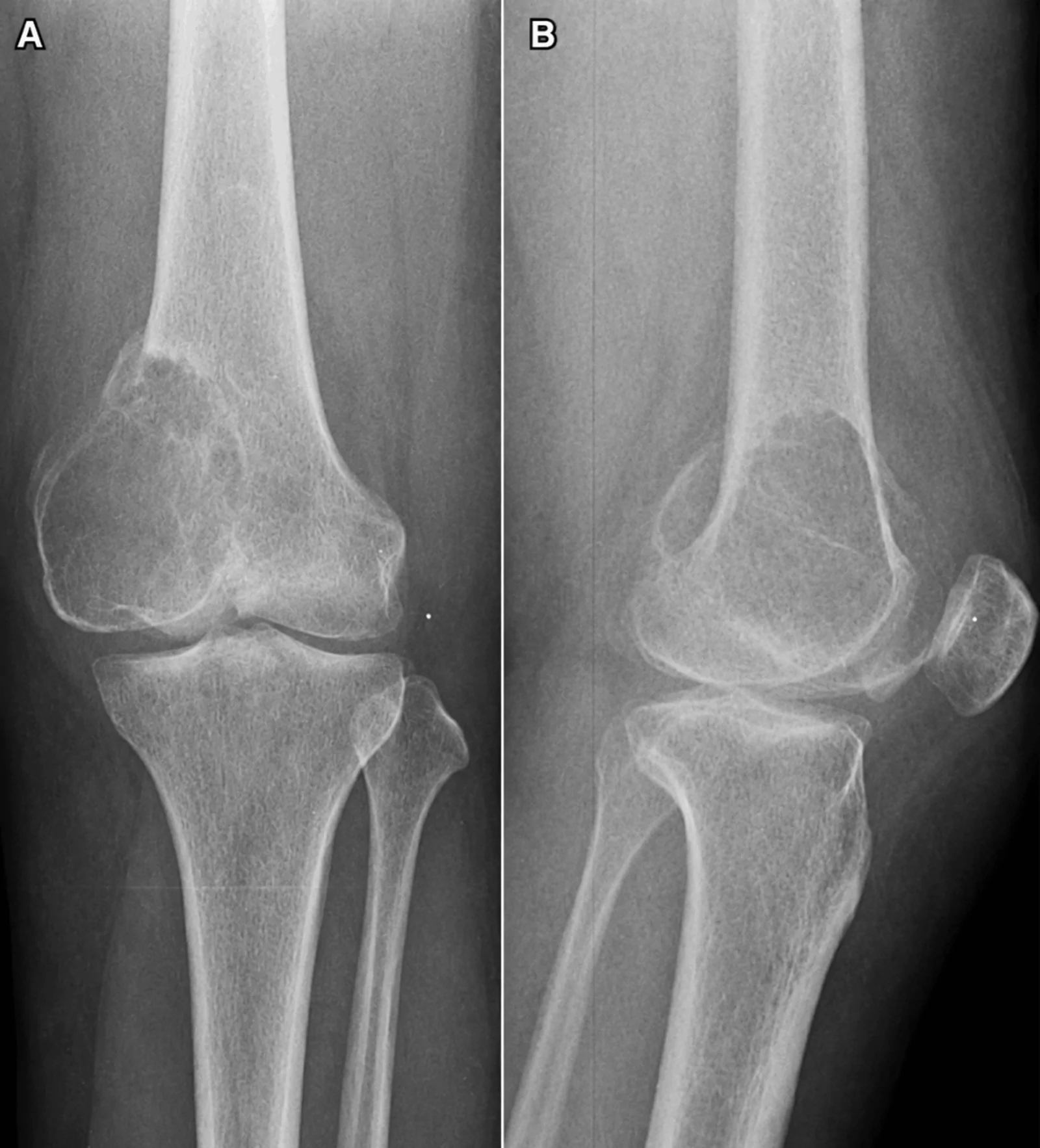
Initial anteroposterior and lateral radiographs of the left knee Anteroposterior (A) and lateral (B) radiographs demonstrating an expansile osteolytic lesion involving the distal femur. The lesion is characterized by cortical thinning, cortical destruction, epiphyseal extension, and absence of matrix mineralization, consistent with an aggressive primary bone tumor.

**Figure 2:**
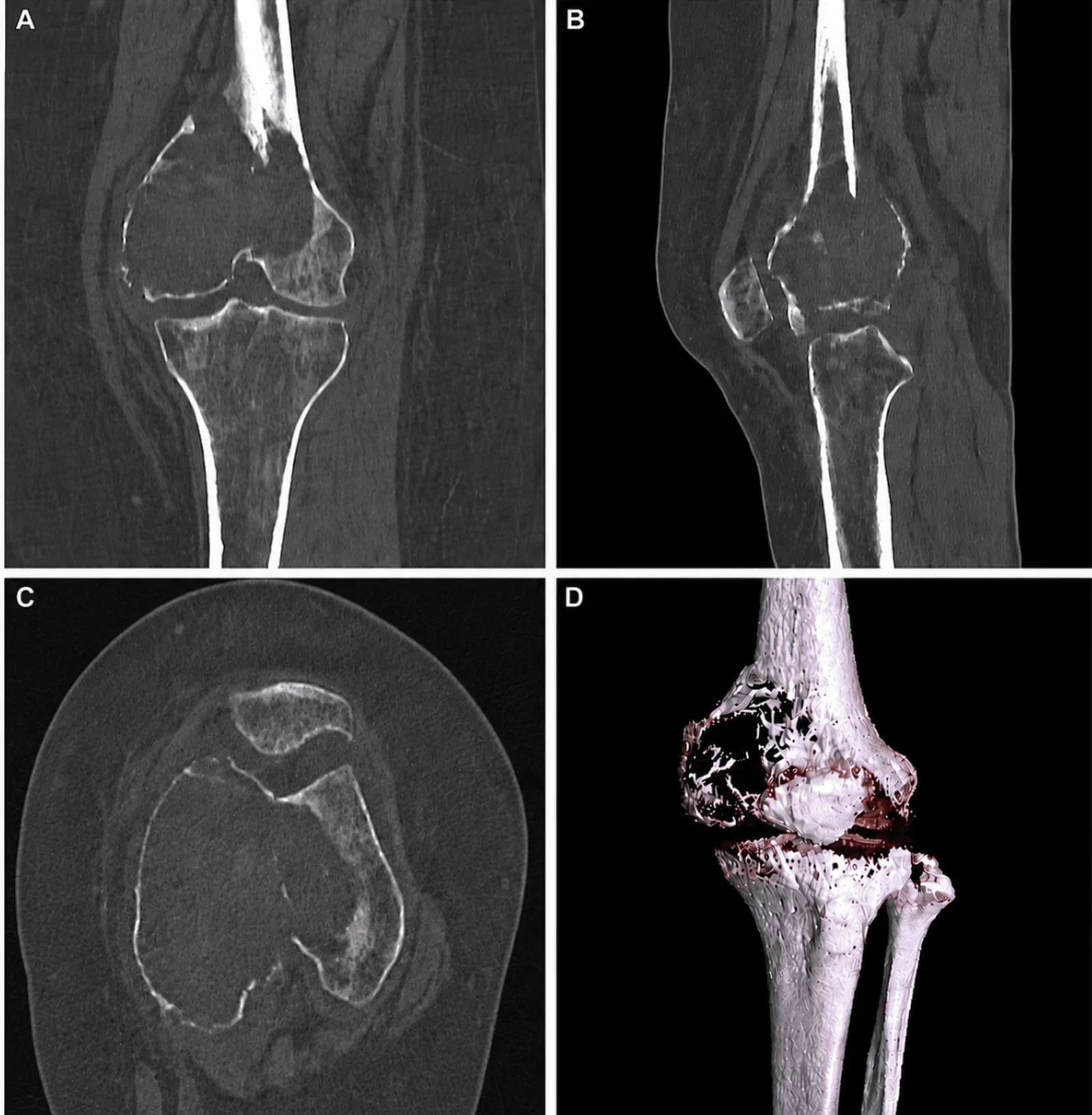
Computed tomography of the distal femoral lesion Coronal (A), sagittal (B), axial (C), and three-dimensional reconstruction (D) computed tomography images of the left distal femur demonstrating an expansile osteolytic lesion with marked cortical thinning, cortical destruction, and extension to the subchondral bone. The lesion involves the distal femoral condyles without evidence of pathological fracture or matrix mineralization.

Open biopsy demonstrated numerous osteoclast-like multinucleated giant cells uniformly distributed within a mononuclear stromal-cell background, confirming GCTB. Based on the extensive cortical destruction and extraosseous extension, the lesion was classified as Campanacci grade III. The defining radiographic characteristics and usual management considerations for each Campanacci grade are summarized in Table 1. Histopathological examination of the resection specimen subsequently confirmed the diagnosis and negative surgical margins (Figures 3, 4).

**Table 1:** Campanacci classification and management considerations for giant cell tumor of bone. Treatment should be individualized and is not determined by radiographic grade alone. Source: Original table created by the authors based on the Campanacci classification described by Campanacci et al. [1]. The table was not reproduced or adapted from the original publication.

Grade	Radiographic Features	Management Considerations
I (latent)	Well-defined lesion with a thin sclerotic rim; intact cortex.	Observation or extended curettage when symptomatic
II (active)	Well-defined margins without a sclerotic rim; cortical thinning and expansion without cortical breach.	Extended curettage with local adjuvants and reconstruction.
III (aggressive)	Cortical destruction with extraosseous soft-tissue extension.	Individualized treatment: extended curettage in selected cases or wide resection when joint-preserving surgery is not feasible.

**Figure 3:**
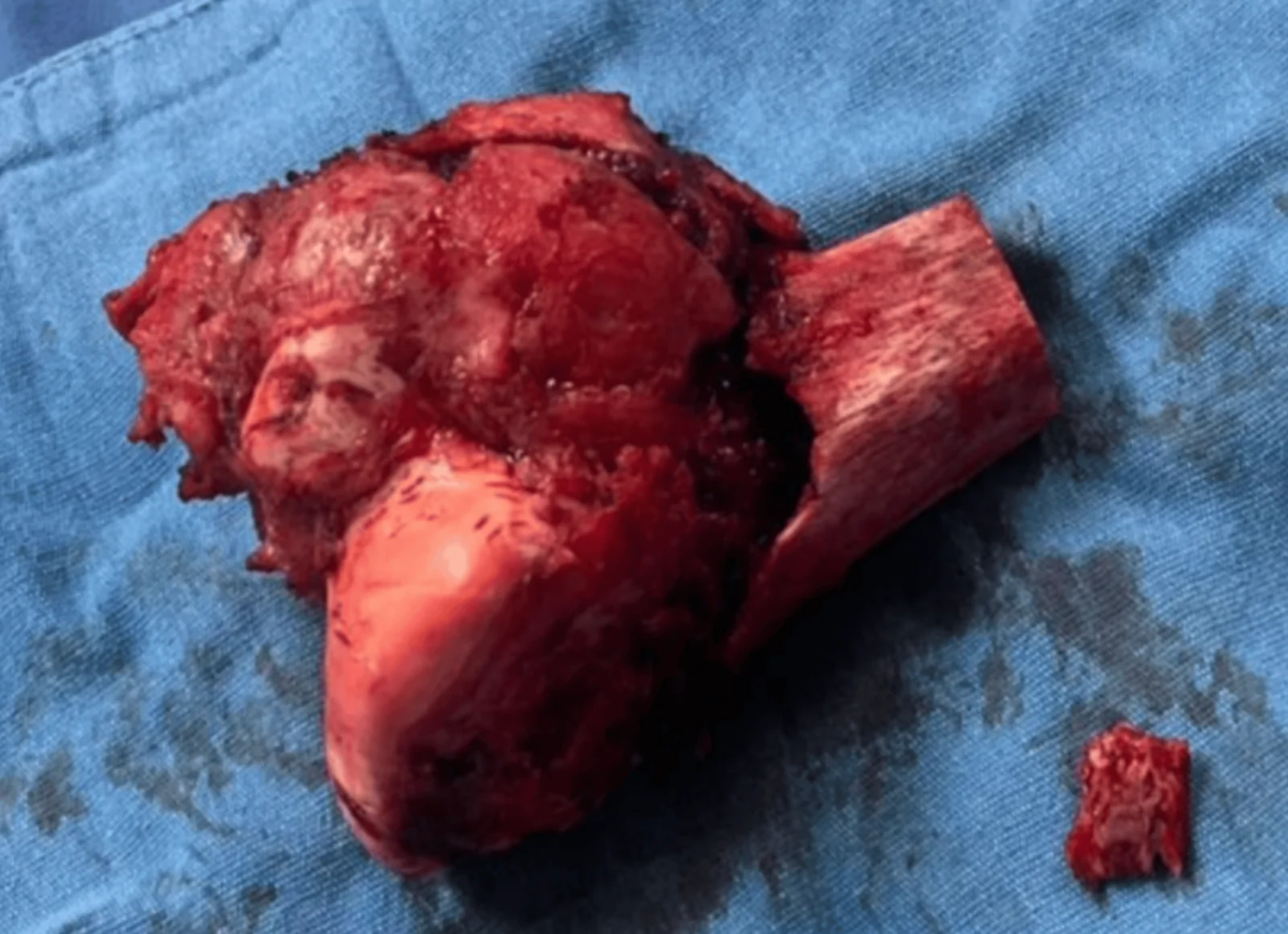
Gross pathological specimen following wide en bloc resection of the distal femur. The specimen demonstrates an expansile epimetaphyseal giant cell tumor with cortical destruction and extension to the subchondral bone.

**Figure 4:**
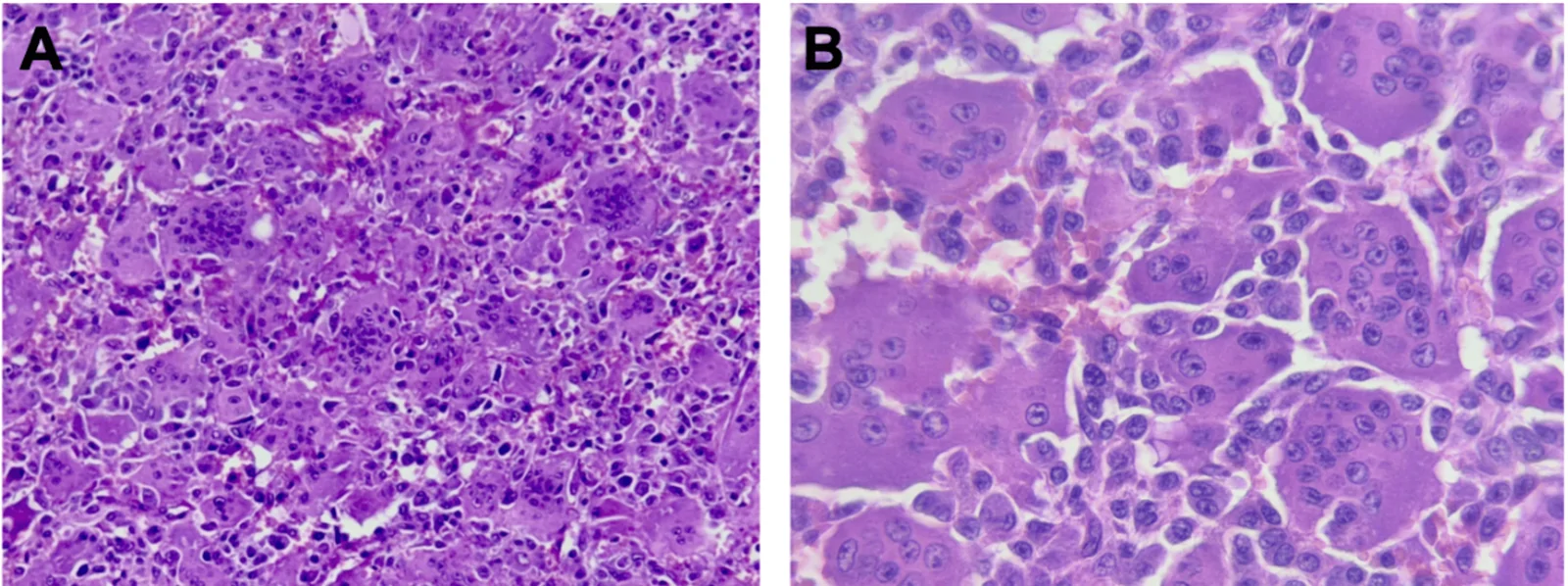
Histopathological examination of the resected specimen. (A) Low-power hematoxylin and eosin (H&E) image showing the tumor–bone interface (original magnification, ×10). (B) High-power H&E image demonstrating numerous osteoclast-like multinucleated giant cells within a background of mononuclear stromal cells, confirming the diagnosis of giant cell tumor of bone (GCTB) (original magnification, ×40).

Therapeutic intervention

After multidisciplinary evaluation, limb-salvage surgery was selected because extensive cortical destruction, involvement of both femoral condyles, and articular compromise made intralesional treatment unsuitable. Through a medial approach, the distal femur was resected en bloc with oncological margins, including both condyles, the metaphyseal region, adjacent soft tissue, and the medial meniscus. The femoral medullary canal was sequentially prepared, and reconstruction was performed using a cemented modular distal femoral megaprosthesis incorporating a rotating-hinge knee articulation (Figure 5). Prosthetic alignment, limb length, and joint stability were verified before definitive implantation.

**Figure 5:**
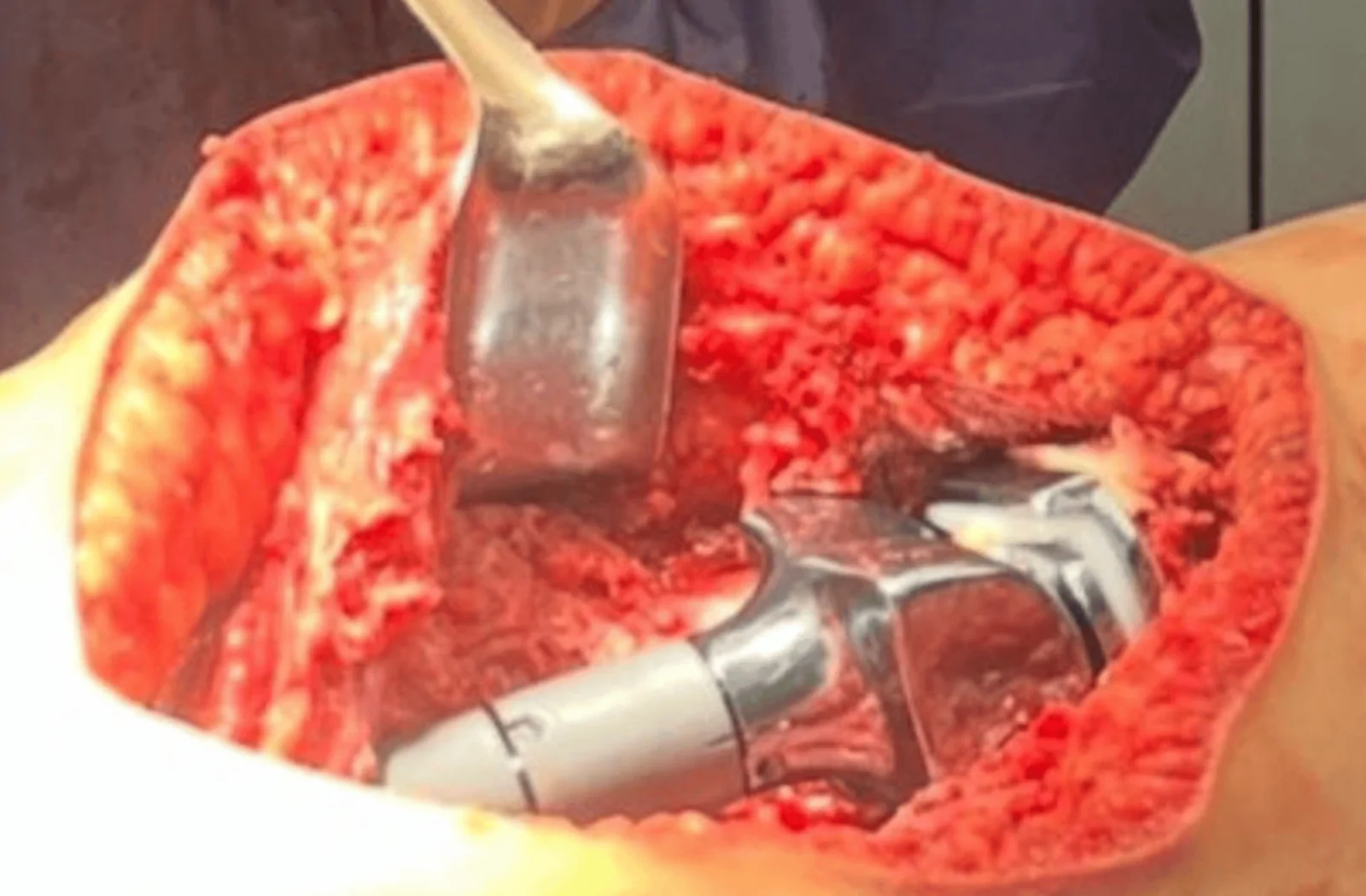
Intraoperative megaprosthetic reconstruction. Following wide en bloc resection of the distal femur, reconstruction was performed using a cemented modular distal femoral megaprosthesis. The image demonstrates the assembled prosthetic components, restoration of limb length and alignment, and reconstruction of the knee joint before definitive soft-tissue closure.

Follow-up and outcomes

The postoperative course was uneventful. During the first 48 hours, monitoring focused on hemodynamic stability, wound surveillance, and pain control; thromboprophylaxis was administered. Rehabilitation began on postoperative day 3 with supervised active and passive knee range-of-motion exercises, quadriceps strengthening, and progressive weight-bearing ambulation using a four-point walker, advanced according to pain tolerance and functional recovery. At three weeks, the incision had healed completely and the sutures were removed. Radiographs showed appropriate alignment and stable fixation of the megaprosthesis without fracture, loosening, or dislocation (Figure 6), and rehabilitation was advanced according to institutional protocol.

**Figure 6:**
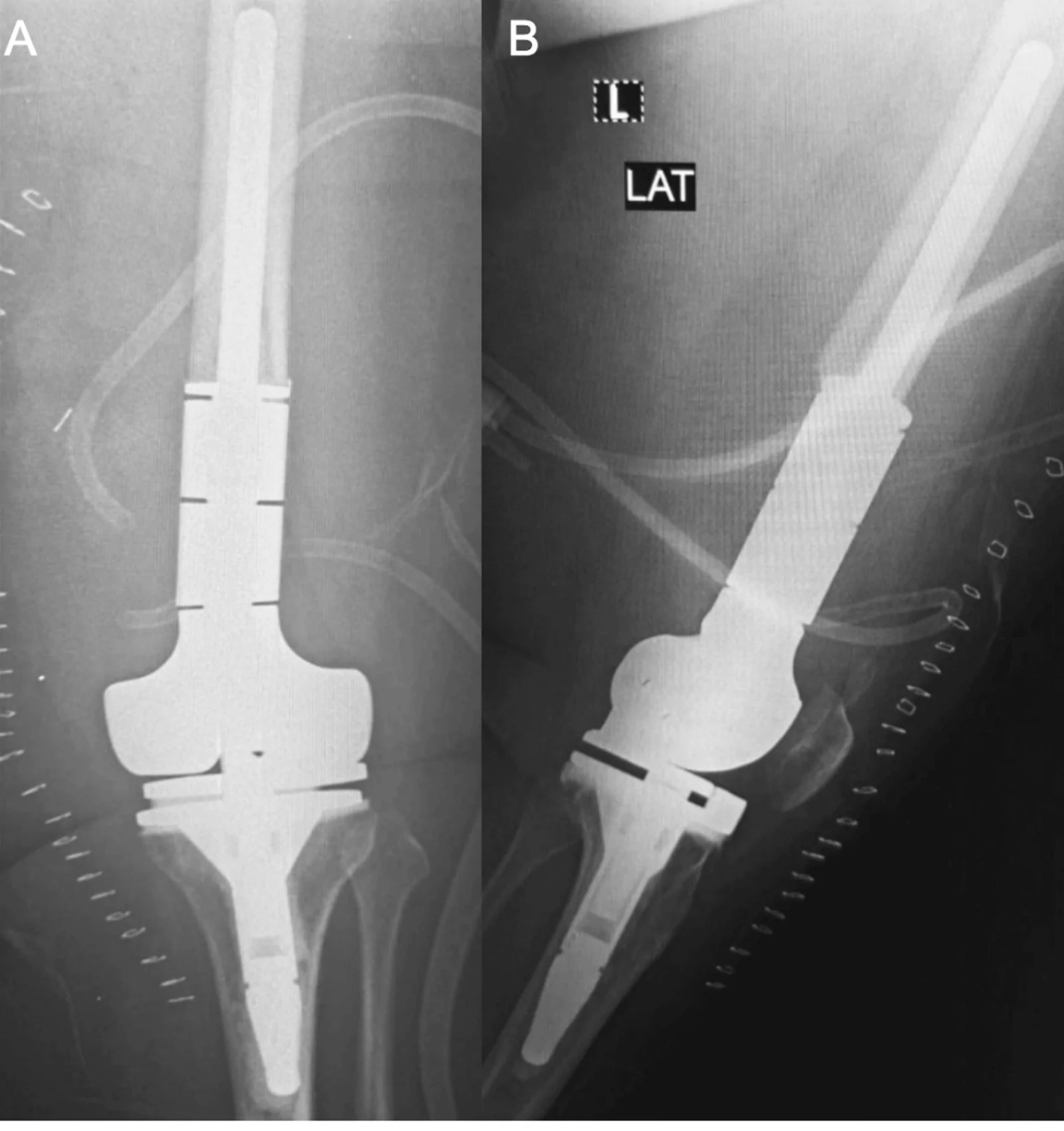
Postoperative radiographs following megaprosthetic reconstruction. Postoperative anteroposterior (A) and lateral (B) radiographs demonstrating satisfactory alignment and fixation of the cemented modular rotating-hinge distal femoral megaprosthesis following wide en bloc resection. No immediate postoperative complications are evident.

At the 12-month follow-up, the patient achieved a Musculoskeletal Tumor Society (MSTS) score of 26/30 (87%), demonstrating substantial pain relief and satisfactory functional recovery, with radiographs confirming stable prosthetic fixation and clinical and imaging surveillance showing no evidence of local recurrence or distant metastasis. The clinical course is summarized, and the individual MSTS domain scores are detailed in Table 2.

**Table 2:** Clinical course and Musculoskeletal Tumor Society functional assessment at the 12-month follow-up. GCTB: Giant cell tumor of bone; MSTS: Musculoskeletal Tumor Society.

Time point/domain	Clinical event or assessment	Key findings/score
CLINICAL COURSE		
Two years before presentation	Symptom onset	Progressive left knee pain, distal-thigh swelling, and functional limitation.
Initial evaluation	Imaging and biopsy	Distal femoral osteolytic lesion; open biopsy confirmed Campanacci grade III GCTB.
Definitive treatment	Surgery	Wide en bloc distal femoral resection and cemented modular rotating-hinge megaprosthetic reconstruction.
Early postoperative period	Recovery	Close monitoring, thromboprophylaxis, pain control, and supervised rehabilitation without immediate complications.
Three-week follow-up	Early assessment	Complete wound healing with stable implant alignment and fixation.
Twelve-month follow-up	Final assessment	Stable prosthesis without local recurrence or distant metastasis.
MSTS ASSESSMENT AT 12 MONTHS		
Pain	Functional domain	4/5
Function	Functional domain	5/5
Emotional acceptance	Functional domain	4/5
Supports	Functional domain	4/5
Walking ability	Functional domain	4/5
Gait	Functional domain	4/5
Total MSTS score	Overall functional outcome	26/30 (87%)

## Discussion

The Campanacci classification remains widely used because it reflects radiographic aggressiveness and assists surgical planning [1]. Grade I and II lesions are generally managed with extended intralesional curettage and local adjuvants. In grade III disease, cortical destruction and extraosseous extension may increase the risk of residual disease and recurrence after intralesional treatment, making wide resection appropriate when joint preservation is not feasible [4,7-9]. In this patient, destruction of both femoral condyles, articular compromise, and soft-tissue extension supported wide resection to obtain negative margins while preserving the limb. Published studies demonstrate that surgical management and recurrence risk vary according to tumor extent, anatomical location, and the feasibility of achieving adequate margins (Table 3).

**Table 3:** Clinical factors supporting wide en bloc resection and megaprosthetic reconstruction.

Clinical factor	Patient finding	Surgical implication
Campanacci grade	Grade III	Supported consideration of wide en bloc resection.
Cortical involvement	Extensive cortical destruction	Adequate local control was unlikely with intralesional curettage.
Articular involvement	Both distal femoral condyles affected	Joint preservation was not feasible.
Soft-tissue extension	Present	Increased concern for residual disease after intralesional treatment.
Postresection defect	Large segmental defect	Required modular distal femoral megaprosthetic reconstruction.

Reconstruction after distal femoral resection is technically demanding because limb alignment, knee stability, and early function must be restored without compromising oncologic principles. A cemented modular distal femoral megaprosthesis provides immediate structural stability and permits early rehabilitation after extensive periarticular resection [5,9-11]. At 12 months, our patient had a Musculoskeletal Tumor Society (MSTS) score of 26/30, stable fixation, and no evidence of local recurrence or distant metastasis [8].

Denosumab, a receptor activator of nuclear factor-kappa B ligand inhibitor, may promote peripheral bone formation and facilitate surgery in selected patients. However, concerns remain regarding residual neoplastic stromal cells and recurrence after discontinuation, and definitive surgical management remains central for resectable Campanacci grade III lesions [6,12-14]. Treatment should be individualized according to tumor extent, anatomical location, articular involvement, anticipated morbidity, and the feasibility of obtaining oncologically adequate margins.

This report is limited by its single-patient design and 12-month follow-up, which are insufficient to assess late recurrence, distant metastasis, and long-term implant survival. Nevertheless, the early result illustrates the role of wide resection and modular endoprosthetic reconstruction when extensive periarticular destruction precludes joint-preserving surgery [15].

## Conclusions

Campanacci grade III GCTB of the distal femur requires individualized treatment that balances oncologic control with limb function. In this case, wide en bloc resection followed by cemented modular distal femoral megaprosthetic reconstruction achieved negative margins, stable fixation, and good early function without local recurrence at 12 months. Longer surveillance is required to determine durable disease control and implant survival.

## References

[1] Campanacci M, Baldini N, Boriani S, Sudanese A (1987). Giant-cell tumor of bone. J Bone Joint Surg Am.

[2] McDonald DJ, Sim FH, McLeod RA, Dahlin DC (1986). Giant-cell tumor of bone. J Bone Joint Surg Am.

[3] Sobti A, Agrawal P, Agarwala S, Agarwal M (2016). Giant cell tumor of bone: an overview. Arch Bone Jt Surg.

[4] Balke M, Schremper L, Gebert C (2008). Giant cell tumor of bone: treatment and outcome of 214 cases. J Cancer Res Clin Oncol.

[5] Nagano A, Urakawa H, Tanaka K, Ozaki T (2022). Current management of giant-cell tumor of bone in the denosumab era. Jpn J Clin Oncol.

[6] Ng VY, Davidson DJ, Kim EY, Pollack SM, Conrad Iii EU, Jones RL (2014). The multidisciplinary management of giant cell tumor of bone. Expert Rev Anticancer Ther.

[7] Montgomery C, Couch C, Emory CL, Nicholas R (2019). Giant cell tumor of bone: review of current literature, evaluation, and treatment options. J Knee Surg.

[8] Enneking WF, Dunham W, Gebhardt MC, Malawar M, Pritchard DJ (1993). A system for the functional evaluation of reconstructive procedures after surgical treatment of tumors of the musculoskeletal system. Clin Orthop Relat Res.

[9] Aoude A, Nikomarov D, Perera JR (2023). Giant cell tumour of bone. Bone Joint J.

[10] Jeys LM, Kulkarni A, Grimer RJ, Carter SR, Tillman RM, Abudu A (2008). Endoprosthetic reconstruction for the treatment of musculoskeletal tumors of the appendicular skeleton and pelvis. J Bone Joint Surg Am.

[11] Chakarun CJ, Forrester DM, Gottsegen CJ, Patel DB, White EA, Matcuk GR Jr (2013). Giant cell tumor of bone: review, mimics, and new developments in treatment. Radiographics.

[12] Myers GJ, Abudu AT, Carter SR, Tillman RM, Grimer RJ (2007). Endoprosthetic replacement of the distal femur for bone tumours: long-term results. J Bone Joint Surg Br.

[13] Sim FH, Choong PFM, Weber KL (2011). Master Techniques in Orthopaedic Surgery: Orthopaedic Oncology and Complex Reconstruction. Master Techniques in Orthopaedic Surgery: Orthopaedic Oncology and Complex Reconstruction.

[14] He H, Zeng H, Luo W, Liu Y, Zhang C, Liu Q (2019). Surgical treatment options for giant cell tumors of bone around the knee joint: extended curettage or segmental resection?. Front Oncol.

[15] Jha Y, Chaudhary K (2023). Giant cell tumour of bone: a comprehensive review of pathogenesis, diagnosis, and treatment. Cureus.

